# Radiologically Isolated Syndrome: 5-Year Risk for an Initial Clinical Event

**DOI:** 10.1371/journal.pone.0090509

**Published:** 2014-03-05

**Authors:** Darin T. Okuda, Aksel Siva, Orhun Kantarci, Matilde Inglese, Ilana Katz, Melih Tutuncu, B. Mark Keegan, Stacy Donlon, Le H. Hua, Angela Vidal-Jordana, Xavier Montalban, Alex Rovira, Mar Tintoré, Maria Pia Amato, Bruno Brochet, Jérôme de Seze, David Brassat, Patrick Vermersch, Nicola De Stefano, Maria Pia Sormani, Daniel Pelletier, Christine Lebrun, on behalf of the Radiologically Isolated Syndrome Consortium (RISC) and Club Francophone de la Sclérose en Plaques (CFSEP)

**Affiliations:** 1 University of Texas Southwestern Medical Center, Department of Neurology & Neurotherapeutics, Clinical Center for Multiple Sclerosis, Dallas, Texas, United States of America; 2 University of Istanbul, Department of Neurology, Cerrahpasa School of Medicine, Istanbul, Turkey; 3 Mayo Clinic College of Medicine, Department of Neurology, Rochester, Minnesota, United States of America; 4 Mt. Sinai School of Medicine, Department of Neurology, Radiology and Neuroscience, New York, New York, United States of America; 5 Barrow Neurological Institute, Department of Neurology, Phoenix, Arizona, United States of America; 6 Cleveland Clinic Lou Ruvo Center for Brain Health, Department of Neurology, Las Vegas, Nevada, United States of America; 7 MS Center of Catalunya Cemcat and Magnetic Resonance Unit, Vall d′Hebron Hospital, Barcelona, Spain; 8 University of Florence, Department of Neurology, Florence, Italy; 9 Centre Hospitalo Universitaire Bordeaux, Bordeaux, France; 10 Centre Hospitalo Universitaire Strasbourg, Strasbourg, France; 11 Centre Hospitalo Universitaire Purpan, Toulouse, France; 12 Centre Hospitalo Universitaire Salengro, Lille, France; 13 University of Siena, Department of Medicine, Surgery & Neuroscience, Siena, Italy; 14 University of Genoa, Department of Health Sciences (DISSAL), Genoa, Italy; 15 Yale University, Departments of Neurology and Diagnostic Radiology, New Haven, Connecticut, United States of America; 16 Hôpital Pasteur, Service de Neurologie, Nice, France; National Institutes of Health, United States of America

## Abstract

**Objective:**

To report the 5-year risk and to identify risk factors for the development of a seminal acute or progressive clinical event in a multi-national cohort of asymptomatic subjects meeting 2009 RIS Criteria.

**Methods:**

Retrospectively identified RIS subjects from 22 databases within 5 countries were evaluated. Time to the first clinical event related to demyelination (acute or 12-month progression of neurological deficits) was compared across different groups by univariate and multivariate analyses utilizing a Cox regression model.

**Results:**

Data were available in 451 RIS subjects (F: 354 (78.5%)). The mean age at from the time of the first brain MRI revealing anomalies suggestive of MS was 37.2 years (y) (median: 37.1 y, range: 11–74 y) with mean clinical follow-up time of 4.4 y (median: 2.8 y, range: 0.01–21.1 y). Clinical events were identified in 34% (standard error = 3%) of individuals within a 5-year period from the first brain MRI study. Of those who developed symptoms, 9.6% fulfilled criteria for primary progressive MS. In the multivariate model, age [hazard ratio (HR): 0.98 (95% CI: 0.96–0.99); *p* = 0.03], sex (male) [HR: 1.93 (1.24–2.99); *p* = 0.004], and lesions within the cervical or thoracic spinal cord [HR: 3.08 (2.06–4.62); *p* = <0.001] were identified as significant predictors for the development of a first clinical event.

**Interpretation:**

These data provide supportive evidence that a meaningful number of RIS subjects evolve to a first clinical symptom. An age <37 y, male sex, and spinal cord involvement appear to be the most important independent predictors of symptom onset.

## Introduction

The radiologically isolated syndrome (RIS) was first introduced in 2009 [1] to define a relevant cohort of individuals routinely encountered in clinical practice who are at risk for future demyelinating events. Formal criteria were generated to enhance the specificity for accurate classification such that risk factors for clinical and radiological progression could be appropriately identified. Even prior to the introduction of RIS criteria [1], longitudinal clinical data from healthy individuals with incidentally identified anomalies suggestive of multiple sclerosis (MS) were described [2], followed by a proposed variant with similar phenomenology [3]. Subsequent to these early reports, advances have been made in the characterization of RIS subjects [4]–[9] and in our understanding of risk factors for initial symptom development [10]–[12].

However, a current challenge in the global application of established criteria for RIS involves the accurate classification of subjects with incidentally identified anomalies highly characteristic for MS, in comparison to those categorized in medical parlance as possessing “unidentified bright objects” or non-specific T2-hyperintensities commonly identified in migraine headache patients [13] that fulfill MS spatial dissemination requirements [14]. Efforts to increase the available data that improve upon the characterization of RIS and risk for symptom manifestation are not only essential to prevent over diagnosis, but to enhance medical counseling, surveillance recommendations, and future research strategies.

This investigation represents an analysis of retrospectively acquired, non-standardized, multi-national data ascertained from the Radiologically Isolated Syndrome Consortium (RISC) in an effort to estimate the risk for an initial clinical event and understand important risk factors for symptom manifestation.

## Methods

### Ethics Statement

This investigation was approved by following regulatory authorities and ethics committees: Committee on Human Research (University of California, San Francisco, California, U.S.A.), Mayo Clinic Institutional Review Board (Mayo Clinic, Rochester, Minnesota, U.S.A.), Institutional Review Boards of Mt. Sinai School of Medicine (Mt. Sinai School of Medicine, New York, New York, U.S.A.) and the Barrow Neurological Institute (Barrow Neurological Institute, Phoenix, Arizona, U.S.A.), Institutional Review Board of Vall d′Hebron Research Institute (MS Center of Catalunya Cemcat, Barcelona, Spain), Institutional Review Board for Human Subjects (University of Florence, Florence, Italy), Institutional Review Board of the University of Siena (University of Siena, Siena, Italy), Istanbul University Cerrahpasa School of Medicine Ethical Committee for Clinical Research and Studies (University of Istanbul, Istanbul, Turkey), and the Comité de Protection des Personnes (Hôpital Pasteur, Nice, France). Written informed consent was acquired from all study subjects.

### Subjects

Retrospectively identified subjects fulfilling 2009 criteria for RIS [1], originating from 22 clinical sites within 5 countries, with initial brain MRI studies revealing incidental anomalies suggestive of demyelinating disease dated >1990 were included. Data originated from existing RIS cases within centers and newly identified RIS cases arising from requested consultations and referrals from community and academic centers. Table 1 summarizes the inclusion and exclusion criteria.

**Table 1:** Inclusion and exclusion criteria for study subjects.

Inclusion criteria	Exclusion criteria
RIS subjects of all ages	MRI scan date <1990
Incidental anomalies identified on MRI of the brain or spinal cord with the primary reason for the acquired MRI resulting from an evaluation of a condition other than MS	Incomplete medical history or radiological data
CNS white matter anomalies meeting the following criteria:	History of remitting clinical symptoms consistent with multiple sclerosis lasting > 24 hours prior to CNS imaging with anomalies suggestive of MS
a. Ovoid, well-circumscribed, and homogeneous foci with or without involvement of the corpus callosum	
b. T2-hyperintensities measuring >3 mm^2^ and fulfilling 3 or 4 Barkhof Criteria for dissemination in space	
c. CNS anomalies not consistent with a vascular pattern	
MRI anomalies do not account for clinically apparent impairments	CNS MRI anomalies are better accounted for by another disease process

In all subjects, demographic characteristics, family history of MS, detailed historical and current clinical data, key episodes in their RIS course, comprehensive neurological examination results, structural neuro-imaging of the brain, and non-standardized blood test results (i.e. sedimentation rate, anti-nuclear antibody, vitamin B12 level, homocysteine and methylmalonic acid, rheumatoid factor, anti-cardiolipin antibody screen, thyroid function studies, serum angiotensin-converting enzyme, and Lyme disease titers) for the evaluation of other medical conditions that could explain the observed lesions on brain MRI were available. Imaging of the spinal cord and other para-clinical studies (i.e. cerebrospinal fluid (CSF) analysis, optical coherence tomography, etc.) were variable amongst individuals and within participating centers.

### Neuro-Imaging Studies

In all cases, the original imaging studies on hard films or within. DICOM files were reviewed to confirm the presence of brain anomalies. All study subjects possessed one or more MRI studies of the brain that fulfilled dissemination in space (DIS) criteria for RIS [1]. Images originated from non-uniform protocols from differing MRI units and magnetic field strengths (1.5 or 3.0 Tesla (T)) and were performed at multiple academic, academic affiliated, and community institutions. All examinations included T1 and T2-weighted spin-echo sequences in multiple planes of view (axial, coronal, and sagittal) with or without the administration of gadolinium. Spinal cord imaging protocols were also heterogeneous with T1 and T2-weighted spin-echo sequences in axial and sagittal planes of view available (with or without gadolinium) and short tau inversion recovery (STIR) sequences accessible for more recent imaging studies.

For all MRI studies reviewed, abnormalities within the brain or spinal cord were initially identified by a neuroradiologist on the formal interpretation and subsequently examined and verified by an MS specialist at each clinical site to ensure DIS MRI criteria were met [1]. A qualitative (i.e. geographical location within the brain or spinal cord, and morphology of lesions) and quantitative (i.e. number of T2-foci, presence or absence of gadolinium enhancement) analysis of the available brain and spinal cord imaging studies was performed.

Brain MRI studies were assessed for the presence of lesions in the supratentorial and infratentorial regions, with specific attention to brainstem structures and the presence of gadolinium enhancement. Lesions within the cervical and thoracic spinal cord were deemed significant utilizing previously reported criteria [12].

### Procedures

The primary outcome for this investigation was to assess the impact of demographic, clinical, and radiological metrics on the time to the first clinical event, defined as the development of an acute neurological episode localized to the optic nerve, brainstem, cerebellum, spinal cord, or long sensory or motor tracts, lasting >24 hours followed by a period of symptom improvement or the onset of a clinical symptom (e.g. leg weakness) with the temporal profile revealing at least a 12-month progression of neurological deficits, across different groups. Multiple sclerosis specialists and site principal investigators were responsible for confirming relapses. Quality assurance measures were performed on all subjects with symptom manifestation to confirm documented neurological events by evaluating the clinical description, localization within the CNS, and, when available, the Expanded Disability Status Scale (EDSS) score.

Subgroups were defined based on demographic factors (i.e. age at the time of RIS, sex, ethnicity, etc.), baseline brain and spinal cord MRI characteristics, CSF profiles, and exposure to approved disease-modifying treatments (DMTs) for MS.

### Statistical Analysis

All calculations and statistical analyses were performed using SPSS for Windows, version 16.0 (SPSS, IBM, Armonk, New York, U.S.A.).

Means with standard deviations (SD) were acquired to summarize the demographic, clinical and radiological data, when appropriate.

The time from RIS diagnosis to the first acute or initial symptom resulting in a progressive clinical course was estimated utilizing Kaplan-Meier survival analyses. Log-rank tests were used to compare survival data between groups at univariate analysis. Multivariate Cox regression models were created to assess the independent predictive value of demographic characteristics (i.e. sex, ethnicity, age at the time of RIS diagnosis), clinical data (i.e. MS family history, exposure to approved DMTs for MS, CSF profiles), and imaging data (i.e. total number of lesions contained within the infratentorial, juxtacortical, and periventricular regions on brain MRI, the presence of gadolinium enhancing lesions throughout the follow-up period, geographical distribution of lesions, involvement of the spinal cord) on the time to the first symptomatic event. The association of each covariate with time to the first clinical symptom was quantified by hazard ratios (HR) along with their 95% confidence intervals (CI).

A *p* value <0.05 was considered significant.

## Results

A total of 456 RIS subjects were identified. Table 2 summarizes the cases by center and country of origin. Five subjects with neuro-imaging studies dated before 1990 were excluded, resulting in 451 cases available for the primary analysis.

**Table 2:** Participating Centers within the Radiologically Isolated Syndrome Consortium (RISC) Research Network and corresponding contribution of new and previously published RIS cases by region.

	New Cases (n = 264)	Previously Reported (n = 187)
**United States of America (n = 205)**		
University of California, San Francisco, San Francisco, California (n = 92)	n = 21	n = 71
Mayo Clinic Medical Center, Rochester, Minnesota (n = 30)	n = 21	n = 9
Mt. Sinai Medical Center, New York, New York (n = 34)	n = 34	–
Barrow Neurological Institute, Phoenix, Arizona (n = 49)	n = 49	–
		
**France (n = 149)**		
**Club Francophone de la Sclérose en Plaques**		
Centre Hospitalo Universitaire Pasteur, Nice, France (n = 51)	n = 23	n = 28
Centre Hospitalo Universitaire Pontchaillou, Rennes, France (n = 4)	n = 2	n = 2
Centre Hospitalo Universitaire Purpan, Toulouse, France (n = 9)	n = 6	n = 3
Centre Hospitalo Universitaire Salengro, Lille, France (n = 25)	n = 13	n = 12
Centre Hospitalo Universitaire Clermont, Clermont-Ferrand, France (n = 2)	n = 1	n = 1
Centre Hospitalo Universitaire Central, Nancy, France (n = 9)	n = 3	n = 6
Centre Hospitalo Universitaire Besançon, Besançon, France (n = 4)	n = 2	n = 2
Centre Hospitalo Universitaire Strasbourg, Strasbourg, France (n = 8)	n = 5	n = 3
Centre Hospitalo Universitaire tripode, Bordeaux, France (n = 10)	n = 7	n = 3
Centre Hospitalo Universitaire Nantes, Nantes, France (n = 7)	n = 3	n = 4
Centre Hospitalo Universitaire Timone, Marseille, France (n = 3)	n = 3	–
Fondation Rothschild, Paris, France (n = 11)	n = 5	n = 6
Centre Hospitalo Universitaire Reims, France (n = 3)	n = 3	–
Centre Hospitalo Universitaire Montpellier Nimes, France (n = 3)	n = 3	–
		
**Italy (n = 24)**		
University of Siena, Siena, Italy (n = 12)	–	n = 12
University of Florence, Florence, Italy (n = 12)	–	n = 12
		
**Turkey (n = 44)**		
University of Istanbul, Istanbul, Turkey	n = 31	n = 13
		
**Spain (n = 29)**		
MS Center of Catalunya Cemcat, Vall d′Hebron Hospital, Barcelona, Spain	n = 29	–

The study cohort was principally comprised of women (F: 354 (78.5%)) who were white (86%). The mean age when the first abnormal MRI occurred was 37.2 years (y) (median: 37.1 y, range: 11–74 y) with mean clinical follow-up time of 4.4 y (median: 2.8 y, range: 0.01–21.2 y). A positive family history for MS was observed in 10.0% of the study group. A total of 31 subjects had EDSS scores >0.0 on the initial baseline examination (Table 3) resulting from the identification of disc pallor on funduscopic examination, mild alterations in visual acuity, mild deficits in vibratory sensation, mild urinary complaints or constipation, depression, fatigue, or pyramidal function system scores >0.0 due to weakness secondary to peripheral neurological disorders. CSF profiles were obtained in 67% (300/451) of all RIS cases. Abnormal CSF profiles were identified in 64.7% (194/300) of samples acquired. The baseline characteristics of the core study cohort are summarized in Table 3.

**Table 3:** Baseline demographic, clinical and radiological data from the entire RIS study cohort.

Cohort (n = 451)	Values	Missing, n (%)
Mean age at RIS, y (median, range)	37.2 (37.1, [11–74]	0 (0)
		
Female, n (%)	354 (78.5%)	0 (0)
		
Ethnicity, n (%)		22 (4.9)
White	387 (85.8)	
African American	6 (1.33)	
Hispanic	17 (3.8)	
Middle Eastern	15 (3.2)	
Asian/Pacific Islander	4 (0.9)	
		
Family History for MS, n (%)	41 (9.9)	38 (8.4)
		
Mean clinical follow-up time, y (median, range)	4.4 (2.8, [0.01–21.1])	2 (0.4)
		
EDSS Score (%)		0 (0)
0	420 (93.1)	
1	23 (5.1)	
1.5	6 (1.3)	
2	2 (0.4)	
		
Abnormal CSF profile*, n (%)	194 (64.7)	151 (33.5)
		
Exposure to DMT, n (%)	73 (17.3)	29 (6.4)
		
Presence of Gd+ lesions, n (%)	108 (28.3)	70 (16)
		
Presence of periventricular lesions, n (%)	440 (98.7)	5 (1.1)
		
Presence of infratentorial lesions, n (%)	137 (30.4)	5 (1.1)
		
Presence of juxtacortical lesions, n (%)	400 (90.1)	7 (1.6)
		
Presence of spinal cord lesions lesions, n (%)	135 (35.2)	68 (15.1)

Reasons for the initial brain MRI were diverse (Table S1). Imaging on the spinal cord was ordered at the discretion of the referring or treating physician at each study site with cervical (326; 72.3%) and thoracic (171; 37.9%) MRI studies performed. Table 3 summarizes the radiological characteristics of the study cohort.

**Figure 1:**
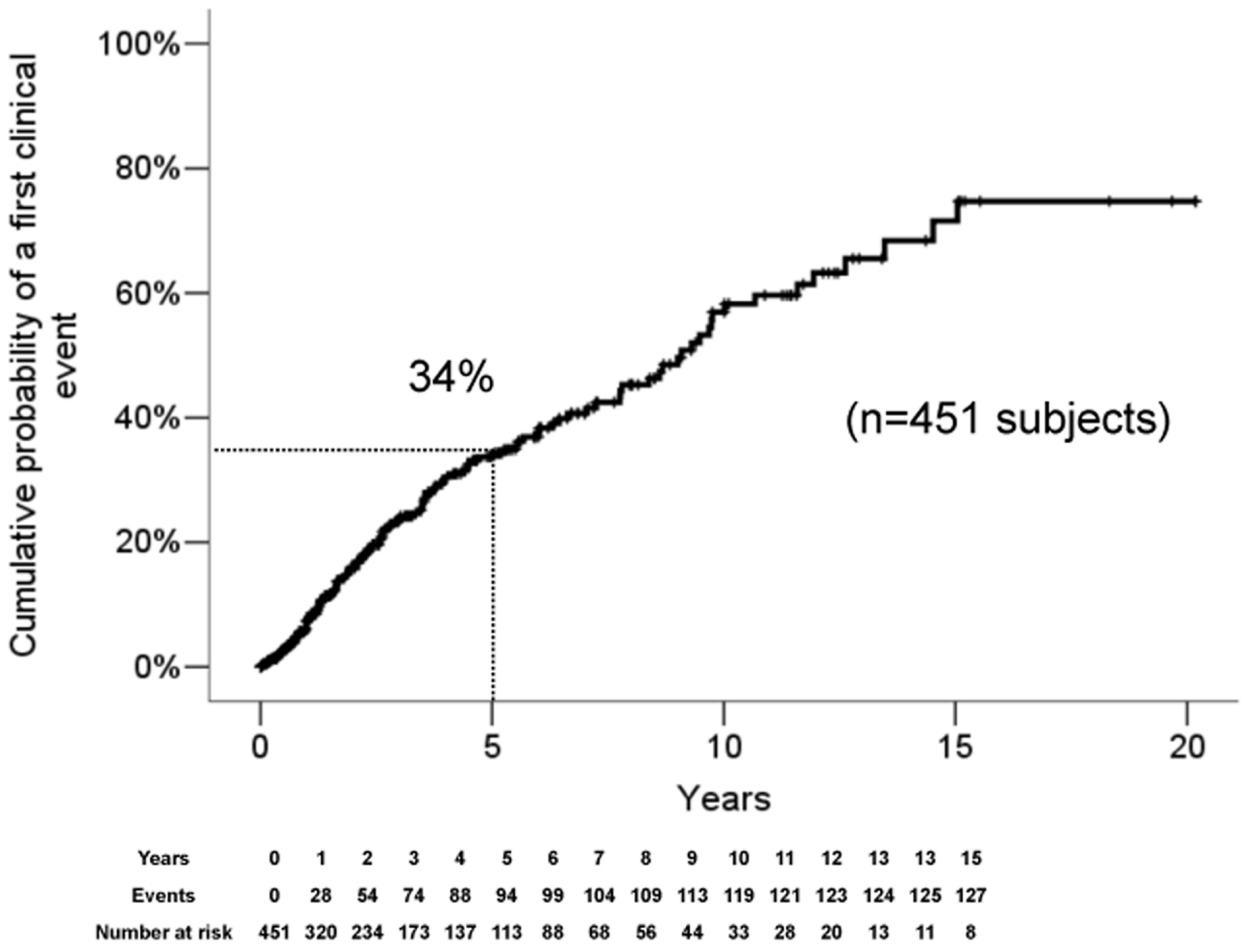
Kaplan-Meier survival analysis with the endpoint of time to the first acute or progressive event at 5-years for the entire RIS cohort.

The 5-year observed first acute or progressive clinical event rate on the whole study population was 34% (standard error (SE) = 3%) (Figure 1). In all subjects experiencing clinical events, symptoms appeared to be consistent with a demyelinating event. The results of the Kaplan-Meier survival analyses did not reveal different temporal profiles of evolution to the first symptomatic event (*p* = 0.40) between all centers over the course of follow-up from RIS diagnosis. In addition, no significant differences related to the reason that induced the MRI with RIS evidence were detected (*p for heterogeneity = 0.76*).

Of the 451 cases included in the analysis, 14 individuals who, after experiencing their first clinical symptom, experienced at least 12-months of progressive neurological decline during the entire follow-up period and fulfilled criteria for primary progressive MS [15] (median age = 43.8 y (interquartile range: 40.7–57.3), sex (male) (50%), white (92.7%)). A total of 9 cases were identified within the first 5 years from the initial MRI demonstrating the incidental anomalies, representing 9.6% of all subjects experiencing a first event.

**Figure 2:**
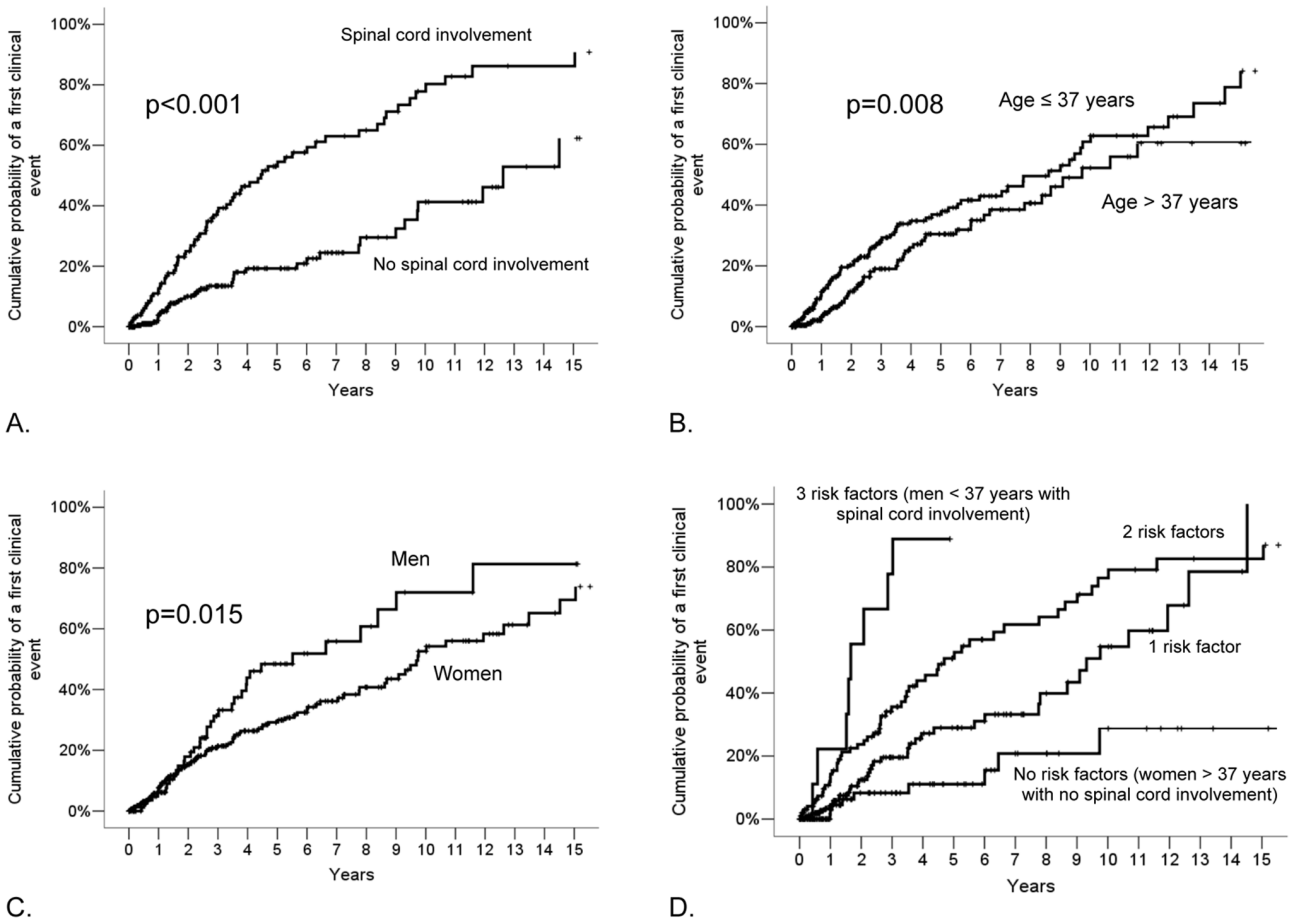
Kaplan-Meier survival analysis with the endpoint of time to a first clinical event by (A) the presence of spinal cord lesions, (B) age at first MRI demonstrating anomalies suggestive of demyelinating disease, (C) sex, and (D) stratified based on the presence of 0, 1, 2, or 3 risk factors.

Results of the univariate and multivariate Cox regression models are reported in Table 4. The following variables were included in the multivariate analysis: age, sex, positive MS family history, ethnicity, abnormal CSF, presence of periventricular lesions, presence of infratentorial lesions, presence of juxtacortical lesions, cervical or thoracic spinal cord lesion, and contrast enhancement on the initial MRI revealing the incidental anomalies highly suggestive of MS. A younger age at RIS diagnosis was associated with an increased risk of developing an initial symptomatic event [hazard ratio (HR): 0.98 (95% CI: 0.96–0.99); *p* = 0.03] with an estimated risk of developing an event decreasing by 2% for every year additional year of age. In addition, the presence of either a cervical or thoracic spinal cord lesion [HR: 3.08 (95% CI: 2.06–4.62); *p*<0.001] constituted significant predictors for the development of a first clinical event (adjusted for Center and date of RIS MRI). The 5-year risk of developing a first clinical event for RIS subjects with both spinal cord involvement and younger age was 58% (SE = 7%). Also, men had a higher risk compared to women [HR: 1.93 (95% CI: 1.24–2.99); *p* = 0.004]. Further stratification of risk within the spinal cord revealed the independent importance of both cervical [HR: 2.02, (95% CI: 1.10–3.73, *p* = 0.02] and thoracic spinal cord [HR: 2.23 (95% CI: 1.20–4.13), *p* = 0.01] involvement, that were retained together in a multivariate model. In the multivariate model, ethnicity, a family history significant for MS, CSF profile, lesion location (i.e. cerebellum and brainstem), and presence of gadolinium enhancement on the baseline MRI scan were not identified as predictors for a first clinical event. Figures 2A–C demonstrate Kaplan-Meier survival curves for these independent risk factors, and risk stratified by the presence of any single risk factor, or the concomitant presence of multiple risk factors (Figure 2D).

**Table 4:** Cox regression models containing univariate and multivariate analyses of factors related to time to the first acute or progressive clinical event.

Variable	n	Univariate Analysis* HR (95% C.I.)	*p*	Multivariate Analysis* HR (95% C.I.)	*p*
Age	451	0.97 (0.96–0.99)	<0.001	0.98 (0.96–0.99)	0.009
Sex (Male)	451	1.64 (1.10–2.44)	0.015	1.93 (1.24–2.99)	0.004
Positive Family MS History	451	2.20 (1.31–3.70)	0.003		
Ethnicity	451		0.99		
Abnormal CSF§	300	1.78 (1.11–2.87)	0.017		
Periventricular lesions presence	446	0.84 (0.21–3.90)	0.8		
Infratentorial lesions presence	446	1.26 (0.88–1.83)	0.2		
Juxtacortical lesions presence	444	0.94 (0.50–1.77)	0.84		
Cervical or thoracic spinal cord lesion	383	3.26 (2.18–4.86)	<0.001	3.09 (2.06–4.62)	<0.001
Contrast enhancement on RIS MRI	381	1.09 (0.70–1.69)	0.70		

*Adjusted for Center and date of RIS diagnosis.

HR  =  hazard ratio; CSF  =  cerebrospinal fluid.

§ =  IgG index >0.7 or the presence of >2 unique oligoclonal bands within the CNS.

In 16.2% (73/451) of RIS subjects, approved DMTs for MS were introduced prior to the development of a first clinical episode with a mean treatment duration of 3.2 y (SD: 3.1 y, range: 0.1–11 y). We identified subjects treated with interferon b-1a (30 mcg weekly), interferon beta-1b, glatiramer acetate, fingolimod, interferon beta-1a (44 mcg thrice weekly) and natalizumab, with a few individuals exposed to more than one treatment. The 5-year risk of developing a first clinical event for RIS subjects who were exposed to DMTs was 45% (SE = 7%) as compared to 31% (SE = 3%) for RIS subjects who did not receive treatment.

## Discussion

The challenge within RIS involves the accurate identification of subjects and valid predictors for a first attack. Defining risk is associated with limitations: i). MRI scans are acquired randomly and not during or immediately following a neurological episode, ii). disease duration is less clear given the absence of a definable clinical event, and iii). the temporal profile of radiological evolution and brain plasticity in such early forms of demyelinating disease is uncertain. Most importantly, there is a risk of the improper classification of subjects by the over application of DIS criteria, resulting in the incorporation of individuals with subclinical brain anomalies originating from another disease process [13], [14], [16].

Data from this retrospective study of RIS cases from the largest existing multinational cohort, in regions of moderate to high MS prevalence [17], suggest that a meaningful amount of subjects evolve to a first acute or progressive clinical symptom. A younger age at RIS identification, sex (men), and involvement of the spinal cord, were identified as most relevant. Risk also appeared to be higher with the presence of multiple risk factors.

In MS, age is inversely related to the time to disability levels by shortening the latency time for attaining disability milestones [18], [19]. Similar outcomes are also observed in men [19]. Increasing age appeared to be important in reducing the risk for symptom development, a plausible observation given a greater degree of disease activity in younger patient groups, suggesting an opportune age window for symptom development [20]. One would anticipate an increase in lesion burden with advancing chronological age, yet the opposite trend was appreciated suggesting possible differences in the time of disease onset, an imbalance between the extent of injury to the oligodendrocyte or axon and innate compensatory mechanisms during the inflammatory event, or the potential influence of other mediating factors.

Asymptomatic lesions within the cervical or thoracic region were identified to be the strongest predictor for forecasting future clinical events [HR: 3.08 (95% CI: 2.06–4.62); *p*<0.001], affirming our previous report of the importance of spinal cord involvement [12]. The observation of lesions within the spinal cord in RIS epitomizes the clinicoradiologic paradox. Similar discordant experiences are frequently observed in MS patients when evaluating the degree of spinal cord involvement in relation to clinical outcomes [21]. Overall, our current and historical observations of the lack of notable clinical events may be related to an earlier timeframe of disease involvement (and less secondary degeneration) or related to limitations in the imaging techniques utilized as quantitative MRI metrics have recently been better able to classify extent of micro-architectural injury in relation to clinical outcomes [22].

The presence of lesions within the spinal cord, together with additional brain lesions suggestive of MS in young individuals, increases the specificity that these anomalies are due to demyelinating disease, being observed in a limited number of cases in those with other neurological diseases [23]. In a recent investigation of CIS patients without spinal cord symptoms, imaging of the parenchyma was of clinical value diagnostically in facilitating an earlier diagnosis and prognostically in predicting conversion to CDMS [24]. Our results support imaging of the cervical and thoracic spinal cord, having utility in the diagnosis of RIS if the brain MRI study is equivocal or fails to meet DIS criteria, and in defining risk for a future clinical event.

Contrast enhancement and infratentorial involvement on the baseline imaging study, known to be of importance in CIS [25], were not predictive of clinical symptom development. This discordance may be explained by the timing of the MRI scans with CIS patients being scanned during or immediately following an acute neurological event or may be related a higher reserve capacity and recovery innate to RIS subjects. Given this, a higher proportion of MRI relapses may be required, compared to CIS, to surpass the threshold for symptom development.

Treatment in RIS failed to demonstrate a benefit in extending the time to the onset of the first symptomatic event. As treatment decisions were made by referring providers and not study investigators, insights into the decision-making process and attitudes by RIS subjects to receive such interventions, are unclear and may have been biased. This observation is concerning given the lack of scientific data supporting treatment along with the high costs and risks associated with exposure. Despite the extensive existing literature supporting the association of early MS treatment and reduction in clinical attack rates [26]–[28], only a well-structured, and appropriately powered, study would clarify this extremely relevant issue in RIS.

Interestingly, our data revealed a pre-symptomatic phase for subjects fulfilling formal criteria for primary progressive MS [15]. Overall, there is a lack of scientific data regarding the existence and time course of the presymptomatic phase prior to the development of the first progressive symptom with the literature being limited to a single case report [29]. A detailed description of this subgroup will be provided in a separate report.

This work represents the introduction of 264 new RIS cases and the inclusion of 8 new databases within the U.S. and Europe, in addition to the incorporation of previously identified subjects [3], [5], [7], [9], [10], [12]. We believe there is great value in incorporating cases from previously published reports as data were enriched in those without clinical events by the addition of radiological and clinical information acquired during routine re-evaluations. No statistical differences in the probability of a first clinical event were observed between new and previously reported cases. In addition, multiple sensitivity analyses were performed to ensure that one given center was not responsible for driving an observed effect.

The limitations of these data include the retrospective study design and accompanying non-standardized procedures for interval clinical surveillance, clinical evaluations following seminal clinical events, radiological metrics, areas of the CNS being imaged, and time intervals for repeat imaging. Our reported findings may also be impacted by selection bias within participating centers and in countries where MRI use is more prevalent. The differing natural history of MS in regions where subjects were acquired, along with clinical follow-up times of variable length for RIS subjects included within the study cohort, may further limit the generalizability of the observed results. In addition, formalized committees that confirmed the presence of anomalies suggestive of MS on CNS neuroimaging or adjudicated relapses within centers were not employed.

The need for systematically acquired data for improvements in the classification of RIS and generation of risk algorithms are critically important, providing the basis for scientifically supported management, and most importantly, minimizing the amount of improperly classified subjects exposed to unnecessary medical testing, MS treatments, and psychological harm. Caution should be applied in the interpretation of these data given our study limitations and future standardized efforts are needed to verify these findings and help to provide better estimates of risk. In time, new approaches in the treatment of RIS may be developed, being viewed as not only inherently sensible, but appropriate, for those at greatest risk for a seminal clinical event.

## Supporting Information

Table S1 Reasons for the initial brain MRI scan identifying CNS anomalies suggestive of demyelinating disease.
(XLSX)

Table S2 Number of identified cumulative first clinical events and subjects at risk by year, stratified by the presence of spinal cord lesions, for the Kaplan-Meier survival analysis in Figure 2A.
(XLSX)

Table S3 Number of identified cumulative first clinical events and subjects at risk by year, stratified by age, for the Kaplan-Meier survival analysis in Figure 2B.
(XLSX)

Table S4 Number of identified cumulative first clinical events and subjects at risk by year, stratified by sex, for the Kaplan-Meier survival analysis in Figure 2C.
(XLSX)

Table S5 Number of identified cumulative first clinical events and subjects at risk by year, stratified by number of risk factors, for the Kaplan-Meier survival analysis in Figure 2D.
(XLSX)
